# PRISM: A Novel Research Tool to Assess the Prevalence of Pseudobulbar Affect Symptoms across Neurological Conditions

**DOI:** 10.1371/journal.pone.0072232

**Published:** 2013-08-21

**Authors:** Benjamin Rix Brooks, David Crumpacker, Jonathan Fellus, Daniel Kantor, Randall E. Kaye

**Affiliations:** 1 Carolinas Medical Center, University of North Carolina School of Medicine–Charlotte Campus, Charlotte, North Carolina, United States of America; 2 Baylor University Medical Center, Dallas, Texas, United States of America; 3 International Brain Research Foundation, Flanders, New Jersey, United States of America; 4 Neurologique, Ponte Vedra Beach, Florida, United States of America; 5 Avanir Pharmaceuticals, Inc., Aliso Viejo, California, United States of America; Baylor College of Medicine, United States of America

## Abstract

**Background:**

Pseudobulbar affect (PBA) is a neurological condition characterized by involuntary, sudden, and frequent episodes of laughing and/or crying, which can be socially disabling. Although PBA occurs secondary to many neurological conditions, with an estimated United States (US) prevalence of up to 2 million persons, it is thought to be under-recognized and undertreated. The PBA Registry Series (PRISM) was established to provide additional PBA symptom prevalence data in a large, representative US sample of patients with neurological conditions known to be associated with PBA.

**Methods:**

Participating clinicians were asked to enroll ≥20 consenting patients with any of 6 conditions: Alzheimer’s disease (AD), amyotrophic lateral sclerosis (ALS), multiple sclerosis (MS), Parkinson’s disease (PD), stroke, or traumatic brain injury (TBI). Patients (or their caregivers) completed the Center for Neurologic Study−Lability Scale (CNS-LS) and an 11-point scale measuring impact of the neurological condition on the patient’s quality of life (QOL). Presence of PBA symptoms was defined as a CNS−LS score ≥13. Demographic data and current use of antidepressant or antipsychotic medications were also recorded.

**Results:**

PRISM enrolled 5290 patients. More than one third of patients (n = 1944; 36.7%) had a CNS-LS score ≥13, suggesting PBA symptoms. The mean (SD) score measuring impact of neurological condition on QOL was significantly higher (worse) in patients with CNS-LS ≥13 vs <13 (6.7 [2.5] vs. 4.7 [3.1], respectively; *P*<0.0001 two-sample *t*-test). A greater percentage of patients with CNS−LS ≥13 versus <13 were using antidepressant/antipsychotic medications (53.0% vs 35.4%, respectively; *P*<0.0001, chi-square test).

**Conclusions:**

Data from PRISM, the largest clinic-based study to assess PBA symptom prevalence, showed that PBA symptoms were common among patients with diverse neurological conditions. Higher CNS−LS scores were associated with impaired QOL and greater use of antipsychotic/antidepressant medications. These data underscore a need for greater awareness, recognition, and diagnosis of PBA.

## Introduction

Pseudobulbar affect (PBA) is a neurological disorder of emotional expression characterized clinically by frequent, involuntary, and uncontrollable outbursts of laughing and/or crying that are incongruous with or disproportionate to the patient’s emotional state [1]–[6]. PBA occurs secondary to multiple neurological diseases or injury, including stroke, amyotrophic lateral sclerosis (ALS), multiple sclerosis (MS), traumatic brain injury (TBI), Alzheimer’s disease (AD), and Parkinson’s disease (PD), among others [3], [7]. Because of their disruptive and often embarrassing nature, PBA episodes may have socially and occupationally disabling consequences, which are superimposed on the burden of the primary neurological disorder [2], [3], [8]. The pathophysiology of PBA is thought to involve injury to the neurological pathways that regulate affect [5], [9], [10]. PBA has been correlated with brain lesions located primarily in the frontal lobes and descending pathways to the brain stem, basis pontis, and cerebellum, which comprise systems thought to be involved in motor control of emotional expression [5], [10], [11]. The occurrence of PBA symptoms thus appears to be determined largely by the anatomic location of brain lesions, independent of the underlying condition [5].

Symptoms of frequent, excessive, and inappropriate laughing and crying associated with neurological conditions have been noted since at least the late nineteenth century [12] and were extensively described before 1940 [9], [12], [13], [14]. The term pseudobulbar affect was coined by Oppenheim in 1911 to describe “spasmodic explosive bursts of laughter or weeping” [15]. However, many other terms have been used to name such symptoms in patients with neurological conditions, including pathological laughing and crying, emotional lability, emotional incontinence, involuntary emotional expression disorder, and emotionalism [3]. Even though PBA symptoms are rather stereotyped, this variety of nomenclature has complicated efforts to estimate PBA prevalence [5], [7]. PBA is also thought to be under-recognized and undertreated because patients, caregivers, and clinicians are unfamiliar with the disorder [3]. When PBA symptoms are evaluated, they may be mischaracterized as a mood disorder such as depression, although PBA is clinically distinct from mood disorders in terms of duration, character, and context [3], [16]–[18]. Furthermore, the two conditions may both be comorbid, as recent studies show a meaningful incidence of depression or depressive symptoms in patients with PBA [8], [18]–[25].

**Figure 1:**
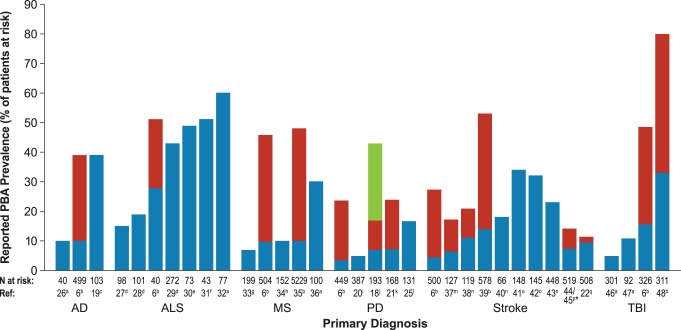
Published PBA symptom prevalence estimates by primary neurological condition. Shading indicates multiple estimates. AD, Alzheimer’s disease; ALS, amyotrophic lateral sclerosis; CNS-LS, Center for Neurologic Study–Lability Scale; MS, multiple sclerosis; PBA, pseudobulbar affect; PD, Parkinson’s disease; PRISM, PBA Registry Series; TBI, traumatic brain injury. ^a^Patient interview; ^b^CNS-LS ≥13 (higher estimate), CNS-LS ≥21, lower estimate; ^c^ Poeck criteria: pathological affect could be mood congruent (emotional lability) or incongruent (pathological laughing and crying); ^d^Retrospective review of hospital or clinic records; ^e^Mailed questionnaire; ^f^Emotional lability questionnaire (ELQ); ^g^Ascertainment method unknown; ^h^Patient interview, Poeck criteria; ^i^Brief questionnaire (uncontrollable laughing/crying when not happy/sad); ^j^CNS-LS ≥13 (highest estimate), CNS-LS ≥17 (middle estimate), Cummings Involuntary Emotional Expression Disorder criteria (lowest estimate); ^k^CNS-LS ≥17 (lower estimate), CNS-LS ≥13 (higher estimate); ^l^Pathological Laughing and Crying Scale (PLACS) ≥10 and score of ≥2 on PLACS items 2 (frequency), 13 (loss of voluntary control), and 18 (distress/embarrassment); ^m^Patient interview House (lower estimate), and Kim (higher estimate) criteria; ^n^Patient interview House criteria; ^o^Patient interview Kim criteria; ^p^Patient interview Kim criteria (lower estimate; n = 516) and modified Kim criteria (patient report only without corroboration from relatives; higher estimate); ^q^Patient interview Kim criteria at hospital admission (lower estimate) and at 3 months (higher estimate) following stroke.

Multiple studies over the past several decades have reported prevalence estimates of involuntary (or pathological) laughing or crying symptoms in populations with specific neurological conditions, including AD, ALS, MS, PD, stroke, and TBI (Figure 1) [7], [18]–[22], [25]–[48] However, these studies used varying criteria for detection and diagnosis of PBA symptoms, and prevalence estimates, even within disease groups, have varied widely (Figure 1) [7]. More recently, an online survey of people registered in the Harris Panel Online (HPOL) who had one of the six neurological conditions listed above estimated that total United States (US) prevalence of persons with PBA symptoms was about 2 million. This estimate was based on screening results in HPOL patients using the Center for Neurologic Study−Lability Scale (CNS-LS), a measure of PBA symptom frequency and severity validated in PBA patients with ALS and MS [32], [49], [50], applied to population data [7]. However, an online survey does not allow for a direct clinical assessment, and the participants may not have been representative of a typical clinic population.

Disease registries are powerful tools for obtaining epidemiologic data [51]–[55]. The PBA Registry Series (PRISM) was established to estimate the prevalence of PBA symptoms in a large representative sample of clinic patients who were diagnosed with common neurological conditions. Additional outcomes of PRISM were to measure the impact of the patient’s neurological condition on quality of life (QOL), and patients’ use of antipsychotic/antidepressant drugs, which are sometimes used to treat PBA symptoms, whether or not they are correctly diagnosed [7], [56].

## Methods

### Overview and Recruitment Goals

**Figure 2:**
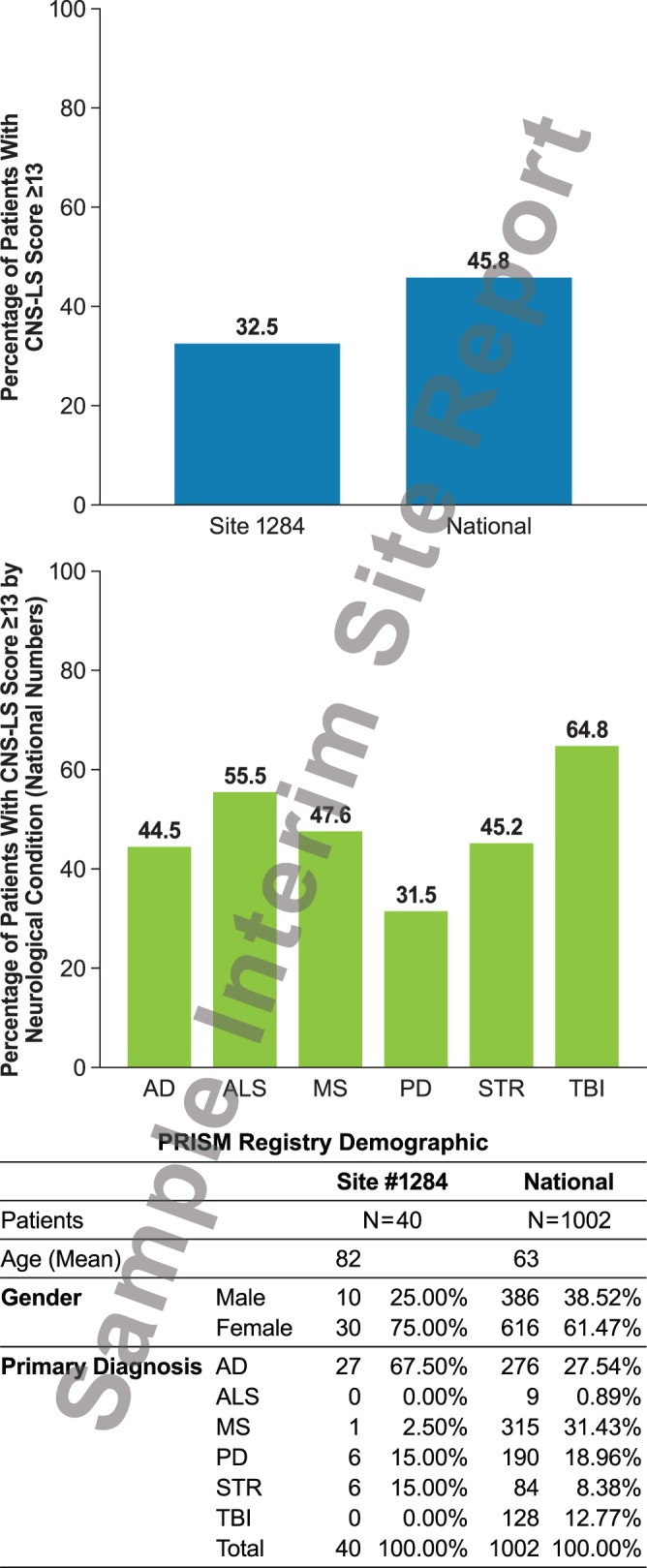
Sample interim PRISM registry report available to activated sites. CNS-LS, Center for Neurologic Study–Lability Scale; PRISM, PBA Registry Series.

PRISM was designed to be a simple patient registry enabling healthcare professionals to capture the prevalence and clinical correlates of PBA symptoms. PRISM aimed to recruit 500 sites nationwide, with each enrolling approximately 20 patients with any of six selected neurological conditions known to be associated with PBA: AD, ALS, MS, PD, stroke, or TBI. The registry was designed to accommodate a total population of up to 10,000 patients. The PRISM protocol was approved by a Central Institutional Review Board" (Compass IRB, Mesa, AZ); all investigators were required to obtain IRB approval from the central IRB or their local IRB before participating. Written informed consent was obtained from each subject or their legally authorized representative prior to data collection. Physicians participating in PRISM had access through the web portal to their own site’s cumulative data, as well as collated national data, so that they could compare their own findings and reports with the collective data reported by other physicians across the US. A sample PRISM report is provided in Figure 2.

### Site Activation Procedures

Licensed healthcare practitioners who managed patients with the above-named neurological conditions and were in good standing with their state review boards were eligible to participate. Investigators registered to participate in PRISM via a centralized web portal [57] and submitted required information and documents to the central IRB for approval. Sites could also work through a local IRB if required. Sites were granted access to begin enrolling patients upon IRB approval.

### Patient Enrollment Procedures

Investigators were instructed to offer the opportunity to participate in PRISM to approximately 20 consenting patients, age 18 or older, with any of the six eligible neurological conditions. Participating patients (or their caregivers, when patients were unable to do so because of cognitive or other disabilities) completed a data capture form, which included an informed consent statement and demographic information including date of birth, sex, primary neurological diagnosis, approximate date of diagnosis, and use of antipsychotic or antidepressant medications. In addition, the patients (or their caregivers) completed the CNS-LS and a QOL measure. The QOL measure rated the response to the question, “How has your neurological condition affected your quality of life?” on a horizontal 11-point integer scale ranging from ‘0′ (not at all) on the left to ‘10′ (strongly affected) on the right. No further screening was conducted for other neurological/psychiatric disorders. The data were then entered by clinic staff into the PRISM registry via the centralized web portal.

### Definition/Detection of PBA Symptoms

The presence of PBA symptoms was defined as a CNS-LS score ≥13; absence of PBA symptoms was defined as a CNS-LS <13. A more restrictive definition was also evaluated using a CNS-LS ≥21. This score is consistent with mean CNS-LS scores of PBA patients who participated in recent clinical trials [7] and has been used in other prevalence surveys to identify a subset of patients likely to have more frequent and severe PBA symptoms [7], [8]. The CNS-LS is the first self-report measure of PBA symptoms to be established and validated; it consists of subscales for laughter (4 items) and for crying (3 items), with each item scored on a 5-point scale (1 = applies never; 5 = applies most of the time) for a total score ranging from 7 (no symptoms) to 35 (maximum) [32], [49]. In patients with ALS (n = 99), a CNS-LS score ≥13 correctly predicted neurologists’ diagnoses of PBA for 82% of patients (sensitivity of 0.84; specificity of 0.81); the CNS-LS also showed good test-retest reliability (0.88) and internal consistency (Cronbach’s α coefficient = 0.87) [32]. In patients with MS (n = 90), a CNS-LS score ≥13 correctly predicted physicians’ diagnoses of PBA for 78% of patients (sensitivity of 0.96; specificity of 0.55), and a CNS-LS score ≥17 correctly predicted 89% of physicians’ diagnoses (sensitivity of 0.94; specificity of 0.83) [49]. The CNS-LS has not been validated in other neurological conditions. In patients with PD, a CNS-LS ≥11 had 100% sensitivity for physician diagnosis of involuntary emotional expression disorder (IEED) but specificity was 48% [18] and thus considered to have poor discriminant validity as a screening tool for PBA.

### Statistical Analysis

Summary statistics were computed for demographic data and disease characteristics for the entire group and within each underlying disease. Between-group differences in categorical variables for participants with and without PBA symptoms were assessed via a chi-square test. Between-group differences in continuous variables were assessed via a two-sample *t*-test. Correlation analyses were performed to evaluate the association between CNS-LS score and patient ratings of the impact of their neurological condition on QOL. Since the stated objective of PRISM was to estimate PBA symptom prevalence, the protocol did not include a plan for inferential statistical analysis of the various outcomes prospectively collected; the statistical analysis plan was constructed prior to any analysis of results.

## Results

Enrollment for the PRISM Registry was closed on September 17, 2012, with 585 registered sites, of which 239 were activated and 173 had enrolled patients. A total of 5290 patients were enrolled, including 1799 (34.0%) patients with AD; 125 (2.4%) with ALS, 1215 (23.0%) with MS, 804 (15.2%) with PD, 757 (14.3%) with stroke, and 590 (11.2%) with TBI. Participant demographics, time since diagnosis of neurological condition, and antipsychotics/antidepressant medication use are presented for the overall group and by underlying neurological condition in Table 1. The mean (SD) patient age in the total population was 65.8 (17.8). Consistent with the neurological conditions evaluated, over half of the patient sample (57.6%) were aged 65 years or older and 39.6% were aged 75 years or older. Mean patient age was lowest in the TBI and MS groups (48.5 and 48.8, respectively) and highest in the PD and AD groups (72.8 and 79.2, respectively). A majority of patients (n = 3184; 60.2%) were female. The time since diagnosis of the primary underlying neurological condition was recorded for only about half of all participants (n = 2677; 50.6%), and was a mean (SD) of 6.7 (8.1) years. A total of 2213 patients (41.8%) reported using at least one antidepressant or antipsychotic medication. Almost one-third of enrolled patients (31.9%) were receiving nontricyclic antidepressant medications (including selective serotonin reuptake inhibitors [SSRIs] and serotonin and norepinephrine reuptake inhibitors [SNRIs]), 13.0% were taking tricyclic antidepressants, and 3.6% were taking antipsychotic medications.

**Table 1:** PRISM patient data.

		Total	AD	ALS	MS	PD	Stroke	TBI
Sample size, n (%)		5290 (100)	1799 (34.0)	125 (2.4)	1215 (23.0)	804 (15.2)	757 (14.3)	590 (11.2)
Patient age, years,	Mean (SD)	65.8 (17.8)	79.2 (9.8)	60.0 (12.8)	48.8 (12.1)	72.8 (10.5)	68.3 (14.7)	48.5 (16.5)
Age group, n (%)	≥65 years	3048 (57.6)	1660 (92.3)	54 (43.2)	112 (9.2)	639 (79.5)	476 (62.9)	107 (18.1)
	≥75 years	2093 (39.6)	1338 (74.4)	15 (12.0)	19 (1.6)	392 (48.8)	284 (37.5)	45 (7.6)
Patient gender, n (%)	Female	3184 (60.2)	1124 (62.5)	47 (37.6)	979 (80.6)	333 (41.4)	408 (53.9)	293 (49.7)
	Male	2106 (39.8)	675 (37.5)	78 (62.4)	236 (19.4)	471 (58.6)	349 (46.1)	297 (50.3)
Years since diagnosis	Sample size	(n = 2677)	(n = 656)	(n = 108)	(n = 785)	(n = 386)	(n = 395)	(n = 347)
	Mean (SD)	6.7 (8.1)	3.7 (3.7)	2.3 (3.2)	9.9 (9.0)	5.3 (6.5)	4.6 (6.0)	10.3 (11.5)
Medication use,* n (%)	Any use	2213 (41.8)	864 (48.0)	50 (40.0)	459 (37.8)	248 (30.8)	296 (39.1)	296 (50.2)
	TCA	689 (13.0)	360 (20.0)	1 (0.8)	34 (2.8)	75 (9.3)	94 (12.4)	125 (21.2)
	nonTCA	1690 (31.9)	592 (32.9)	47 (37.6)	414 (34.1)	178 (22.1)	235 (31.0)	224 (38.0)
	Antipsychotic	190 (3.6)	85 (4.7)	4 (3.2)	31 (2.6)	23 (2.9)	18 (2.4)	29 (4.9)
	Any 2	332 (6.3)	165 (9.2)	2 (1.6)	18 (1.5)	28 (3.5)	47 (6.2)	72 (12.2)
	Any 3	12 (0.2)	4 (0.2)	0 (0)	1 (0.1)	0 (0)	2 (0.3)	5 (0.8)

* Use of antidepressant or antipsychotic medication.

AD, Alzheimer’s disease; ALS, amyotrophic lateral sclerosis; MS, multiple sclerosis; nonTCA, antidepressant other than tricyclic antidepressant; PD, Parkinson’s disease; SD, standard deviation; TCA, tricyclic antidepressant; TBI, traumatic brain injury.

### Prevalence of PBA Symptoms

**Figure 3:**
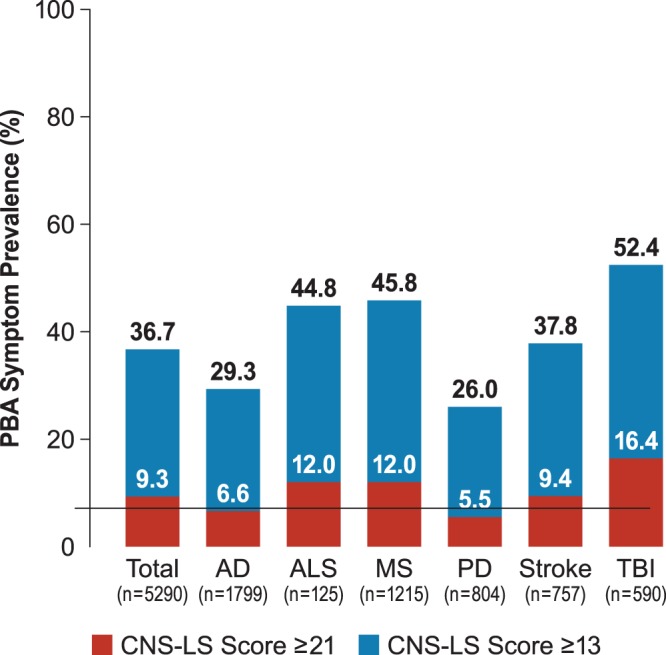
PBA symptom prevalence by CNS-LS threshold. AD, Alzheimer’s disease; ALS, amyotrophic lateral sclerosis; CNS-LS, Center for Neurologic Study–Lability Scale; MS, multiple sclerosis; PBA, pseudobulbar affect; PD, Parkinson’s disease; PRISM, PBA Registry Series; TBI, traumatic brain injury.

Over a third of registry participants (n = 1944; 36.7%) had a CNS-LS score ≥13, suggesting presence of PBA symptoms. The proportion of patients with CNS-LS ≥13 among each neurological condition was lowest for those with PD (26.0%) and highest for those with TBI (52.4%; Figure 3). A total of 492 participants (9.3%) had a CNS-LS score ≥21 (Figure 3). The proportion of patients with CNS-LS ≥21 among the neurological disease groups followed a similar pattern to that seen for the CNS-LS ≥13 threshold and was also lowest for patients with PD (5.5%) and highest for patients with TBI (16.4%) (Figure 3).

A significantly greater proportion of women in the registry than men had CNS-LS ≥13, both in the overall group (41.2% vs. 30.0%; *P*<0.0001) and in each underlying disease state with the exception of ALS and TBI (AD: 33.5% of females vs. 22.4% of males, *P*<0.0001; ALS: 51.1% vs. 41.0%, *P* = 0.2743; MS: 47.8% vs 37.7%, *P*<0.0052; PD: 34.8% vs 19.7%, *P*<0.0001; Stroke: 41.7% vs 33.2% *P* = 0.0171; TBI: 54.3% vs 50.5% *P* = 0.3604).

### QOL Scores

**Figure 4:**
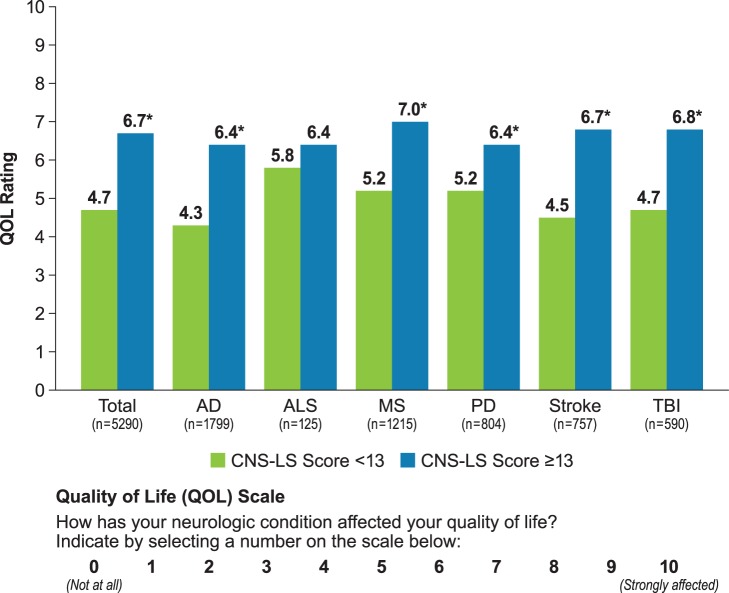
Impact of neurological condition on quality of life by CNS-LS threshold. *P<0.0001 compared with CNS-LS <13; two-sample *t*-test. CNS-LS, Center for Neurologic Study–Lability Scale. Note: Group with CNS-LS ≥21 is a subset of the group with CNS-LS ≥13.

QOL scores are presented by CNS-LS threshold in Figure 4. Among PRISM participants with a CNS-LS score ≥13, the mean (SD) QOL score was significantly higher (worse) than that for patients with CNS-LS <13 (6.7 [2.5] vs. 4.7 [3.1], respectively; *P*<0.0001; Figure 4). Mean QOL scores were also significantly higher for participants with CNS-LS ≥13 within each neurological condition (*P*<0.0001) with the exception of ALS (Figure 4). Additionally, correlation analysis showed an association of higher CNS-LS scores with higher (worse) QOL scores (Pearson’s correlation 0.330; Spearman’s correlation 0.335).

### Medication use by CNS-LS Score

**Figure 5:**
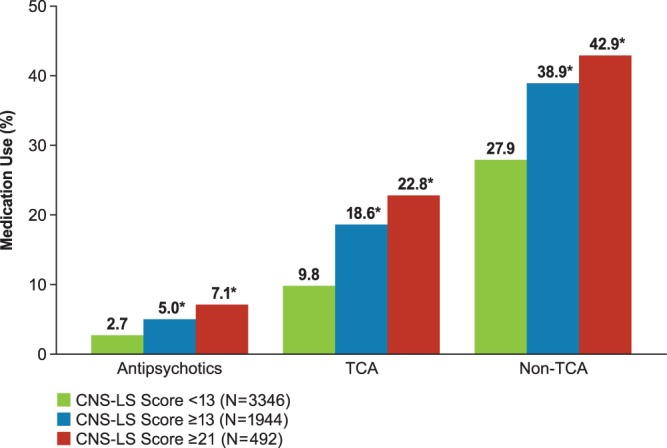
Psychotropic medication use by CNS-LS threshold. *P<0.0001 compared with CNS-LS <13; chi-square test. CNS-LS, Center for Neurologic Study–Lability Scale; TCA, tricyclic antidepressant(s). Note: Group with CNS-LS ≥21 is a subset of the group with CNS-LS ≥13.

The proportion of patients taking at least one antidepressant/antipsychotic medication was significantly higher among those with CNS-LS ≥13 (53.0%) or CNS-LS ≥21 (61.6%) versus those with CNS-LS <13 (35.4%; *P*<0.0001 for both comparisons, chi-square test); differences were noted for each class of medication recorded, as shown in Figure 5.

## Discussion

The PRISM registry is the largest study to date to assess PBA symptom prevalence in a clinical setting across a variety of neurological conditions. This nationwide US study found that 36.7% of recruited patients had a CNS-LS score ≥13, which suggests PBA symptoms. Based on this CNS-LS cutoff, the estimated PBA symptom prevalence is consistent with the prevalence of PBA symptoms (37.5%) found in a similar online survey of 2318 patients/caregivers of patients registered in the HPOL with the same six neurological conditions [7]. The percentage of patients with CNS-LS ≥21 was also consistent in both studies (9.3% in PRISM and 9.4% in the HPOL online survey). The two studies differed in patient selection (clinic patients versus online survey), relative proportion of underlying neurological conditions in the overall study sample, and PBA symptom prevalence in some of the underlying disease states studied. Still, the results do appear to establish the consistency of findings obtained through CNS-LS survey methods.

PBA prevalence studies conducted in specific neurological disorders have generally shown the lowest prevalence estimates in patients with PD and the highest in patients with ALS (Figure 1). TBI is the least well-studied group and has shown the greatest variability in prevalence estimates (Figure 1). Prevalence studies that have primarily utilized patient interviews to diagnose PBA have generally reported lower estimates than studies that based diagnosis on rating scale scores (Figure 1 [see footnotes describing methods]), possibly due to lack of complete diagnostic specificity of rating scales to predict clinical diagnosis, or alternatively to the potential for rating scales to pick up less frequent or less intense symptoms.

Taken together, the data from the registry, the online survey, and existing prevalence literature present increasingly consistent evidence that PBA symptoms are common in patients with diverse neurological diseases. Although the prevalence of PBA may vary across different neurological diseases, the general clinical manifestations of episodes are similar, irrespective of etiology [4]. This consistency would appear to support existing research suggesting that PBA manifestations are determined by the anatomical location of brain lesions, specifically those that disrupt cortico-pontine-cerebellar neural networks that regulate emotional expression [5].

The finding that patients with a CNS-LS ≥13 rated their neurological condition as having a greater negative impact on their QOL is interesting and parallels the findings from a burden of illness (BOI) study conducted as a follow-on to the online HPOL prevalence study discussed above [7], [8]. In the BOI study, respondents with a CNS-LS ≥13 had worse scores for the impact of laughing/crying episodes on QOL and quality of relationships, and had worse scores on all 8 domains and both the mental and physical component summary scores of the 36-item short-form health survey (SF-36), a well-validated measure of general health and well-being [58], compared with respondents with a CNS-LS <13 [8]. The BOI study also found that among survey respondents with CNS-LS ≥13 and involuntary episodes of laughing/crying, these episodes contributed a great deal to or were the main reason for becoming housebound for 24% of these patients, and for being moved to supervised living placement for 9% of these patients.

The presence of inappropriate laughing and crying has been consistently associated with negative health status. A study in stroke survivors (n = 385) found that patients with poststroke emotional incontinence (PSEI) (n = 58; 15.1%) 3 months following the (latest) stroke had worse SF-36 physical and mental component summary scores than patients without PSEI [59]. Diagnosis of PSEI in this study was made on the basis of a psychiatric interview and structured questionnaire and defined as excessive or inappropriate laughing, crying, or both, compared with the premorbid state, on at least 2 occasions following the latest stroke, according to both the patient and one or more cohabitating relative. Another study in patients with PD and other movement disorders that evaluated PBA symptoms using a CNS-LS cutoff score of ≥13 (and also ≥17) showed worse Parkinson’s Disease Questionnaire (PDQ-39) emotional well-being subscores compared with patients without PBA symptoms (CNS-LS <13 or <17) [21].

Psychotropic medication use was also significantly higher in patients with CNS-LS ≥13 versus CNS-LS <13 (Figure 5). It is unclear whether the association between CNS-LS score and medication use denotes an attempt to treat PBA symptoms or treatment of another condition, as the reasons for use of psychotropic medications were not captured.

Significantly more women than men reported PBA symptoms in the PRISM study in each disease group except ALS and TBI. A study by Kim et al in 148 patients evaluated 2–4 months post stroke also found a significant association of female gender with PBA symptoms (post-stroke emotional incontinence) but not post-stroke depression (PSD) [41]. In contrast, most other studies that evaluated potential gender differences have not found an association [20], [22], [25], [30], [34], [36], [37], [42], [45] or have noted that gender disparity was not an independent predictor of PBA on multivariate analysis [18], [44]. Our study did not evaluate other factors that might have accounted for the gender difference noted in those with and without PBA symptoms and it is not clear whether the gender difference is actual or better accounted for by other factors.

Several limitations of the PRISM study must be noted. It should be clearly understood that while the CNS-LS assesses the frequency and severity of the hallmark symptoms of PBA, it does not confer a clinical diagnosis of PBA and has not been validated in all of the neurological conditions studied in PRISM. Despite any diagnostic limitations of the CNS-LS, the results of PRISM suggest a meaningful presence of inappropriate or excessive laughing or crying in patients with these neurological conditions that may be associated with morbidity in terms of impact on QOL and medication use. Uncovering these symptoms is an important first and critical step toward differential diagnosis. The PRISM registry also did not evaluate the prevalence of other neuropsychiatric conditions such as depression or bipolar disorder. This allows for the possibility that some symptoms captured by the CNS-LS (e.g., crying), were indicative of other psychiatric conditions (e.g., depression) or indicative of presence of psychiatric conditions along with PBA. The prevalence of depression in the subcortical dementias can be high. Lifetime depression prevalence in MS patients is estimated to be about 50% [60], annual depression prevalence in ALS patients has been estimated to be about 56% [61], and among PD patients the combined rate of major depression disorder, minor depression, and dysthymia was found to be about 52% [62].

On the other hand, it should also be noted with regard to crying symptoms that there are surprisingly few empirical data supporting a relationship between frequent or labile crying and depression, despite the common (and reasonable) assumption by clinicians that such an association exists [63]–[66]. Indeed, the DSM-IV criteria for diagnosis of major depression do not require changes in either the threshold or intensity of the crying response [64], which are defining indicators of PBA. Nonetheless, because participants were not screened for mood disorders and were not diagnosed clinically, the potential for other etiologies of laughing and crying symptoms cannot be ruled out.

In addition, the QOL question did not measure the direct impact of PBA symptoms on QOL, but the overall impact of the neurological condition on QOL. Therefore, it is not possible to determine whether, or the degree to which, PBA symptoms caused or contributed to QOL impact. Nevertheless, higher CNS-LS scores did correlate with worse QOL.

PRISM did not study all neurological conditions where PBA is known to occur. The study design did not stratify nor weight enrollment relative to prevalence of neurological diseases in the US population. In addition, some sites had a relatively large number of patients enrolled. For example, 7.3% of the entire study population was enrolled by a single site (including 12.8% of AD patients and 11.6% of stroke patients). Similarly, 67% of ALS patients were enrolled by investigators at a single site, and 11% TBI patients were enrolled by a single investigator. It must also be considered that some investigators may not have enrolled consecutive patients or may have selected patients for evaluation because they suspected PBA. For all of these reasons, the prevalence of PBA symptoms observed in this study may not be indicative of the true prevalence of PBA among all patients with neurological conditions. PRISM was a point prevalence study meant to evaluate the overall prevalence of PBA in a typical clinical office setting. While the results can serve to bring awareness to the frequency and impact of PBA symptoms in patients with common neurological conditions, PRISM was not designed to evaluate the clinical course of symptoms over time. Finally, while the statistical analysis plan was not prespecified, the analyses conducted were generally descriptive in nature and the hypotheses tested were fairly broad. Irrespective of the analysis plan, the sheer number of patients assessed, the consistency of results between PRISM and the previous HPOL survey, and the face validity of typical PBA symptoms (scale or no scale), all support the main conclusion: symptoms of excessive or inappropriate laughing and crying are prevalent across these neurological disorders and a PBA diagnosis needs to be entertained in order to properly assess patients with these symptoms.

## Conclusions

The PRISM registry is currently the largest clinic-based study to assess PBA symptoms among neurological disorders known to be associated with PBA. PBA symptom frequency and severity (CNS-LS score) were correlated with impaired QOL. These data underscore a need for greater recognition and diagnosis of PBA, including using targeted questions or screening tools. Further research should be conducted to evaluate the extent to which screening tools such as the CNS-LS confirm actual PBA presence, as well as the extent to which reduction of identified PBA episodes may improve patient outcomes.
